# Dementia and Driving (2nd Cycle Audit)

**DOI:** 10.1192/bjo.2025.10543

**Published:** 2025-06-20

**Authors:** Mohammed Al-Dabbagh, Faquiha Muhammad, Dolapo Odegbaro

**Affiliations:** NHFT, Northampton, United Kingdom

## Abstract

**Aims:** This is round 2 of audit focusing on assessing the compliance of health professionals with the UK law by informing the drivers with dementia about their legal requirement to report their condition to the DVLA and their insurance companies. The aim of this audit is to ensure public safety by adhering to the General Medical Council (GMC) guidance; “Confidentiality: patients’ fitness to drive and reporting concerns to the DVLA or DVA”, as well as the Driving with Dementia or Mild Cognitive Impairment Consensus Guidelines for Clinicians; endorsed by RCPsych and Alzheimer’s Society. This will help ensure public safety and prevent potential accidents or incidents caused by impaired driving.

**Methods:** This is the second cycle of the audit, including patients diagnosed in 2024. First cycle was completed last year for patients diagnosed in 2022. Retrospective data was collected from SystmOne. 40 patients were selected randomly from 807 patients referred to the memory clinic of Watermill resource centre in Berrywood Hospital.

Inclusion Criteria: Patient referred to the service between 1 January–31 December 2024 who were diagnosed with Dementia.

**Results:** The results showed that Compliance with informing patients to report to the DVLA following their diagnosis has improved from 73% to 80%. The compliance with informing patients to report to their insurance companies fell from 45% to 0% in the second cycle. Out of the 40 patients diagnosed with dementia, 34 had a recorded risk assessment. 5 patients were driving at the time of assessment. 3 patients were referred to occupational therapy for a driving assessment. 4 out 5 driving patients were informed they must report to the DVLA (compliance 80%), and 1 out 5 driving patients were informed they must contact their insurance company (compliance 20%). No documented evidence was found about informing the patients about consequence of not reporting to the DVLA and insurance companies. There was no record of medics having to contact the DVLA.

**Conclusion:** Overall, the audit revealed a need for improvement in compliance and documentation. It is recommended that health professionals strictly adhere to their responsibilities in risk assessment and informing drivers with dementia about their legal requirements regarding informing DVLA and insurance companies. Clear documentation should be made using a standard template available.

